# Effectiveness and User Perception of an In-Vehicle Voice Warning for Hypoglycemia: Development and Feasibility Trial

**DOI:** 10.2196/42823

**Published:** 2024-01-09

**Authors:** Caterina Bérubé, Vera Franziska Lehmann, Martin Maritsch, Mathias Kraus, Stefan Feuerriegel, Felix Wortmann, Thomas Züger, Christoph Stettler, Elgar Fleisch, A Baki Kocaballi, Tobias Kowatsch

**Affiliations:** 1 Centre for Digital Health Interventions Department of Management, Technology, and Economics ETH Zurich Zurich Switzerland; 2 Department of Diabetes, Endocrinology, Nutritional Medicine and Metabolism Bern University Hospital University of Bern Bern Switzerland; 3 School of Business, Economics and Society Friedrich-Alexander-Universität Erlangen-Nürnberg Nürnberg Germany; 4 School of Management Ludwig-Maximilians-Universität München Munich Germany; 5 Centre for Digital Health Interventions Institute of Technology Management University of St Gallen St Gallen Switzerland; 6 Department of Endocrinology and Metabolic Diseases Kantonsspital Olten Olten Switzerland; 7 School of Computer Science University of Technology Sydney Sydney Australia; 8 Institute for Implementation Science in Health Care University of Zurich Zurich Switzerland; 9 School of Medicine University of St Gallen St Gallen Switzerland

**Keywords:** hypoglycemia, type-1 diabetes mellitus, in-vehicle voice assistant, voice interface, voice warning, digital health intervention, mobile phone

## Abstract

**Background:**

Hypoglycemia is a frequent and acute complication in type 1 diabetes mellitus (T1DM) and is associated with a higher risk of car mishaps. Currently, hypoglycemia can be detected and signaled through flash glucose monitoring or continuous glucose monitoring devices, which require manual and visual interaction, thereby removing the focus of attention from the driving task. Hypoglycemia causes a decrease in attention, thereby challenging the safety of using such devices behind the wheel. Here, we present an investigation of a hands-free technology—a voice warning that can potentially be delivered via an in-vehicle voice assistant.

**Objective:**

This study aims to investigate the feasibility of an in-vehicle voice warning for hypoglycemia, evaluating both its effectiveness and user perception.

**Methods:**

We designed a voice warning and evaluated it in 3 studies. In all studies, participants received a voice warning while driving. Study 0 (n=10) assessed the feasibility of using a voice warning with healthy participants driving in a simulator. Study 1 (n=18) assessed the voice warning in participants with T1DM. Study 2 (n=20) assessed the voice warning in participants with T1DM undergoing hypoglycemia while driving in a real car. We measured participants’ self-reported perception of the voice warning (with a user experience scale in study 0 and with acceptance, alliance, and trust scales in studies 1 and 2) and compliance behavior (whether they stopped the car and reaction time). In addition, we assessed technology affinity and collected the participants’ verbal feedback.

**Results:**

Technology affinity was similar across studies and approximately 70% of the maximal value. Perception measure of the voice warning was approximately 62% to 78% in the simulated driving and 34% to 56% in real-world driving. Perception correlated with technology affinity on specific constructs (eg, Affinity for Technology Interaction score and intention to use, optimism and performance expectancy, behavioral intention, Session Alliance Inventory score, innovativeness and hedonic motivation, and negative correlations between discomfort and behavioral intention and discomfort and competence trust; all *P*<.05). Compliance was 100% in all studies, whereas reaction time was higher in study 1 (mean 23, SD 5.2 seconds) than in study 0 (mean 12.6, SD 5.7 seconds) and study 2 (mean 14.6, SD 4.3 seconds). Finally, verbal feedback showed that the participants preferred the voice warning to be less verbose and interactive.

**Conclusions:**

This is the first study to investigate the feasibility of an in-vehicle voice warning for hypoglycemia. Drivers find such an implementation useful and effective in a simulated environment, but improvements are needed in the real-world driving context. This study is a kickoff for the use of in-vehicle voice assistants for digital health interventions.

## Introduction

### Background

Type 1 diabetes mellitus (T1DM) is a chronic condition caused by an inability of the pancreas to produce insulin and requires lifelong insulin therapy [1]. Hypoglycemia, also known as low blood glucose, is a frequent and acute complication in patients with T1DM [2,3]. Symptoms range from autonomic reactions such as trembling, anxiety, and hunger (ie, mild hypoglycemia) to neuroglycopenic reactions such as vision impairment, weakness, or cognitive impairments (ie, severe hypoglycemia) [2,4-6]. Hypoglycemia is a major issue in the context of driving: research has shown that hypoglycemia is associated with a higher risk of car mishaps [7-9]. In fact, drivers experiencing hypoglycemia are recommended by the local authorities [10] to stop the car and treat their condition. However, drivers do not always comply with these recommendations [11,12]. Thus, to help reduce hypoglycemia-related car accidents, there should be an effective warning that informs the driver about an upcoming hypoglycemic episode and supports the driver in coping with the situation. Currently, hypoglycemia can be detected and signaled through flash glucose monitoring (FGM) or continuous glucose monitoring (CGM) devices (ie, wearable receivers connected to a sensor inserted in the subcutaneous tissue of the arm or abdomen) [13]. These allow for glucose monitoring by displaying the values either continuously (ie, CGM) or upon active retrieval (ie, FGM) and deliver alerts in the form of a tone or vibration in case of out-of-range values. However, these devices present limitations in the context of driving. For instance, FGM needs to be held close to the sensor to transfer the data from the subcutaneous sensors to the monitoring device, that is, the driver needs to actively engage in a manual gesture to access the glucose value and to look at a visual display moving the focus of attention from the driving task. In contrast, allowing the drivers to receive an alert in a hands-free mode will facilitate warning reception [14] and lower worry associated with driving with T1DM [15]. However, hypoglycemia is known to cause a decrease in attention [2,4-6], thereby challenging the effectiveness of such devices. As 90% of road accidents are caused by human error, the European Commission has set new safety technologies as mandatory equipment for vehicles as of 2022 (eg, driver drowsiness and distraction warnings and speed assistance) [16]. In-vehicle warning systems for impaired driver states, such as fatigue [17], distraction [18], and breath alcohol concentration [19], are increasingly being developed. However, to the best of our knowledge, there is no existing implementation for hypoglycemia. Such technology would be aligned with the “healing car” concept [20], where vehicles become environments promoting well-being for passengers, including ergonomic seats, ambient lighting, relaxation exercises [21], and detection of health-critical states [22]. This concept is still in its early stages, but it may become a standard in car manufacturing in the future. So far, the only attempts of in-vehicle glucose monitoring are either only proof of concept without user validation [23] or conceptual work [24]. However, the online community clearly expressed a need for in-vehicle glucose monitoring and warning [25].

A growing number of automotive companies are introducing voice assistance technology into their products [26,27]. Voice assistants add value not only for the associated consumer experience but also for their greater safety. Indeed, vocal interactions have been observed to be the least cognitively demanding while driving compared with visual and haptic interactions [28,29]. Moreover, voice assistants are increasingly being implemented to deliver digital health interventions [30-33]. Although research is still in its infancy, efforts have been made to develop voice-based conversational agents to monitor and support individuals with chronic diseases such as cancer, cardiovascular diseases, cognitive disorders, or diabetes [30]. Other recent examples include prevention of excessive alcohol consumption [34], health education and monitoring, physical and mental exercise, and nutrition [35]. Furthermore, a voice assistant delivering a warning is a form of proactive behavior initiated by the computer rather than the user [36,37]. In-vehicle voice assistants can provide personalized and adaptive suggestions, but users may ignore proactive behavior if it is inopportune, violates privacy, or distracts from driving [38-40]. However, emergencies are the most suitable context for proactive behavior that violates privacy [39].

### Objectives

Therefore, we investigate the feasibility of an in-vehicle voice warning delivered by a built-in voice assistant to alert and support drivers with T1DM during hypoglycemia. To the best of our knowledge, there have been no investigations on safe and effective in-vehicle hypoglycemia warnings to support drivers with T1DM or on the perception of such technology. Thus, we sought to answer the following research questions (RQs):

- RQ1: How do drivers perceive an in-vehicle voice warning for hypoglycemia while driving?
- RQ2: How effective is an in-vehicle voice warning in prompting drivers to cope with hypoglycemia?

RQ1 refers to the attitude of drivers toward the warning, whereas RQ2 refers to the driver’s compliance behavior once the warning is delivered. Answering these RQs will allow us to conclude on the feasibility of an in-vehicle voice warning for hypoglycemia. To control for individual factors influencing the perception of the warning [41], we also assessed technology affinity.

## Methods

### Study 0: Preliminary Assessment With Healthy Individuals in Simulated Driving

#### Driving Setting

Participants performed the task in a driving simulator (Carnetsoft Inc) with 3 monitors displaying the front, left, and right views. The central monitor also showed the cockpit and navigation arrows. The participants used a steering wheel and pedals (Logitech Driving Force G29) to control the simulator, which was set to automatic (ie, no clutch or gear shifter). The simulator’s computer was connected to a stereo speaker with a subwoofer, which was kept at a constant volume. To control for driving difficulty, 3 environments were used: highway, countryside, and town, with the first and last being the least and most difficult, respectively.

#### In-Vehicle Voice Warning Simulation

Before testing a hypoglycemia voice warning with people with T1DM, we tested the concept of a car voice assistant as an interface between a dedicated monitoring system and the user with healthy participants. As the participants were not affected by hypoglycemia, the first version of the warning was a simulated low fuel warning (“The car needs a refill. Please pull over and turn off the engine”). Although not health related, it signaled an event of reasonable urgency that required safely stopping the car. Note that the participants were informed that this message aimed to ask them to stop the car as soon as possible and that they did not need to look for a gas station.

The warning was simulated using the Wizard-of-Oz method, where the conversational turns produced by the voice assistant were played by the experimenter [42] from a laptop using predefined keyboard keys. The turns were based on the Google Cloud text-to-speech engine, with a de-DE-Wavenet-C voice, a speed of 1.11 times the normal native speed of the specific voice, and a pitch of −1.20 semitones from the original pitch. The experimenter’s computer was connected to the same sound system as the driving simulators so that the voice warning could be heard as part of the driving simulation. No visuals were included.

#### Voice Warning Evaluation Measures

To assess the RQs, we assessed participants’ perception of the warning (self-reported through the modular evaluation of key Components of User Experience [meCUE]; 10 constructs evaluated on a 7-point Likert scale and a general evaluation evaluated on a 10-point scale [43,44]) and participant compliance with the warning (measured by the experimenter manually assessing if the participant would pull over and stop the car following the warning, and reaction time in seconds from the timestamp of the warning to the timestamp of the car fully stopped). As the perception of technology can be influenced by technology-related personality [45], we also measured technology affinity (measured by the Affinity for Technology Interaction [ATI], a 6-point Likert scale [46]). Finally, qualitative feedback was collected informally.

#### Evaluation Procedure

The participants were welcomed, informed about the procedure, and invited to sit in the simulator. The voice assistant introduced itself and invited the participants to familiarize themselves with the setting, including the 3 environments. The training also screened for motion sickness.

In the experimental session, participants drove 12 times, with 4 blocks of 3 drives each, for approximately 5 minutes per drive. The driving environment’s order and starting point varied to minimize habituation. The drive began when the voice assistant prompted participants to start the engine. A timer started to deliver the low fuel warning at either 100 or 200 seconds to add variation and minimize habituation effects. At the end of the session, participants completed the meCUE.

#### Data Analysis

Participants were characterized by sex, age, and driver’s license duration. The ATI was aggregated as a whole, and meCUE items were aggregated per construct. All reports were aggregated across the sample, with mean and SD. Compliance was coded as binary (0=not compliant, 1=compliant) and reported in terms of frequency. Reaction time was aggregated in seconds across participants and phases, with mean and SD.

### Study 1: Assessment With Individuals With T1DM in Simulated Driving

Following the iterative approach described earlier, we conducted 3 exploratory iterations. This study was part of a clinical trial registered at ClinicalTrials.gov (NCT04035993).

#### Driving Setting

The driving setting was the same as in study 0.

#### In-Vehicle Voice Warning Simulation

On the basis of the results of study 0, we adapted the warning to hypoglycemia instead of low fuel, using the fewest conversational turns possible [47]. To ensure that the drivers were available, the voice assistant started with a receptivity check: “May I disturb you?”

We designed the warning based on the guidelines of the Swiss Diabetes Association [10], which recommends taking carbohydrates and stopping the car as soon as signs of hypoglycemia are noticed. To give the driver a sense of autonomy [48], we designed the warning to suggest eating carbohydrates rather than directly engaging in stopping the car. However, if the driver did not have carbohydrates, they were asked to pull over. On the basis of the feedback, we enhanced the voice warning used in the following study to recommend pulling over directly (detailed conversation flow is available in Multimedia Appendix 1).

As in study 0, the warning was simulated with a Wizard-of-Oz method [42], and the turns were generated by recording the same voice. However, to reduce fatigue and cognitive load, we decreased the speed and pitch to 0.93 times the normal speed and −4.8 semitones from the original pitch, respectively. As in study 0, the experimenter would play the turns from a Microsoft Windows laptop using predefined keyboard keys to play prerecorded voice sounds. However, in study 1, the laptop program included a visualization mirrored on a smartphone. The visuals consisted of a blue circle that gradually faded in and out when the voice assistant was speaking. As in study 0, the experimenter’s computer was connected to the same sound system as the driving simulators, so that the voice assistant could be heard as part of the driving simulation.

#### Voice Warning Evaluation Measures

Perception assessment focused on evaluating the voice assistant as a trustworthy driving companion. Specifically, participants completed the Acceptance and Use of Technology (AUT) questionnaire [49,50], the Session Alliance Inventory (SAI) [51], and the Emotional Trust and Competence Trust subscales (henceforth Trust) of the Trust and Adoption questionnaire [52].

To assess technology affinity, participants completed the streamlined scale of the Technology Readiness Index (TRI 2.0) [53]. Items were rated on a 5-point Likert scale (ie, 1=totally disagree, 5=totally agree). We also added a question on whether the participants had previous experience with in-vehicle voice assistants (ie, “Have you already had experience with in-vehicle voice assistants?” with a yes or no answer).

Finally, to obtain qualitative and more in-depth feedback for improvement, we conducted a semistructured interview about their experience with the warning (the interview questions are provided in Multimedia Appendix 2).

#### Evaluation Procedure

The procedure was the same as in study 0, except that participants drove only once for 5 minutes (the evaluation procedure is detailed in Multimedia Appendix 3). Before driving, we ensured that the participants had normal blood glucose levels (5-8 mmol/L).

#### Data Analysis

The sample of participants was characterized by sex, mean age, and mean duration of their driver’s license before the study.

TRI and SAI were aggregated as a whole, and the AUT and Trust items were aggregated per construct. Scores from the negatively formulated questionnaire items were inverted. Previous experience with an in-vehicle voice assistant was reported in terms of frequency. All these reports were aggregated across participants of each iteration, with mean and SD.

To further explain results in perception, they were associated with technology affinity measures. The difference in perception between participants with and without experience with an in-vehicle voice assistant was tested using a 2-sided *t* test, and it was correlated with the TRI constructs using a Pearson test.

Compliance was defined as whether the participant would comply with the warning and was coded as binary (0=did not comply, 1=complied). Reaction time was aggregated in seconds with mean and SD. Compliance behavior was aggregated across participants of each iteration.

Feedback was summarized in positive and negative topics, with a focus on the most prominent suggestions for improvement. Feedback was aggregated across participants of each iteration.

### Study 2: Assessment With Individuals With T1DM in Real-World Driving Undergoing Hypoglycemia

Following the iterative approach described earlier, we conducted 2 exploratory iterations. This study was part of a clinical trial registered at ClinicalTrials.gov (NCT04569630).

#### Driving Setting

Participants drove in Volkswagen Touran on a closed circuit accompanied by a driving instructor. Dual pedals allowed the driving instructor to intervene and stop the car if necessary. The driving environments on the test track corresponded to the environments of the driving simulator used in the previous studies. Straight paths, turns, crossroads, stop signs, and a pedestrian crossing with a doll were used to implement the highway, countryside, and town scenarios. Artificial obstacles (eg, boxes and lines of traffic pylons) were used to simulate the traffic.

#### In-Vehicle Voice Warning Simulation

On the basis of the participant feedback from study 1, we revised the voice warning and addressed low trust ratings by explaining the cause of the warning. We simulated driving behavior as a trigger to detect hypoglycemia while driving, as in the study by Lehmann et al [54]. We created 2 variations of the simplified hypoglycemia notification—one with a statement of the cause (driving behavior) and one without. The final recommendation was reformulated as stricter but less directive than that in study 1.

In the second iteration, we simplified the conversational flow by removing the receptivity check (“May I disturb you?”) and the final recommendation (Multimedia Appendix 1 provides the conversation flow).

We used the Wizard-of-Oz method to simulate the warning, as in studies 0 and 1. We implemented the voice assistant in a smartphone with the same voice as in study 1. However, the experimenter had to control it remotely (outside the car), so we implemented the interaction in a smartphone app controlled by a remote desktop application. The experimenter used the smartphone screen to control the voice warning delivery; therefore, no visualization was included. Because of network-related slowdowns in the remote control, we used a combination of remote control and speech-to-text programing.

#### Voice Warning Evaluation Measures

All measures were the same as in study 1. Reaction time was calculated from the warning onset until the car reached a velocity of 0. In addition, at the end of the experiment, we included a questionnaire item asking which of the 2 types of warning they preferred, that is, the warning including a statement of the cause that triggered the warning or the one without it, or if they would not use either of them.

#### Evaluation Procedure

After welcoming participants and explaining the procedure and simulated voice assistant, the voice assistant introduced itself as an in-vehicle assistant to support drivers with hypoglycemia. The participants then completed a training drive.

The warning was delivered at different stages of hypoglycemia (see the study by Lehmann et al [54]). Drive blocks were defined based on blood glucose levels. In the first phase, the participants drove at normal glucose (5-8 mmol/L). In the second phase, blood glucose level was progressively lowered below the moderate hypoglycemia threshold (3.0 mmol/L) to a target range of 2.0 to 2.5 mmol/L. In the third phase, moderate hypoglycemia was maintained. In the fourth phase, participants drove again with normal blood glucose levels (Multimedia Appendix 3).

To explore the effect of blood glucose level on warning perception and compliance, we delivered a warning at the end of the last drive of each phase. Participants received 2 warnings with an explanation and 2 without, in randomized order.

#### Data Analysis

Data analysis was carried out as in study 1.

### Ethical Considerations

Study 0 was approved by the Ethics Board of ETH Zürich, Switzerland (2019-N-32), and study 1 and study 2 were approved within the context of the HEADWIND study by the cantonal ethics commission of Bern, Switzerland (2020-00685 and 2021-02381, respectively). Study 1 and study 2 are available at ClinicalTrials.gov (NCT04035993 and NCT04569630, respectively). All participants provided written informed consent.

## Results

### Study 0: Preliminary Assessment With Healthy Individuals in Simulated Driving

**Figure 1:**
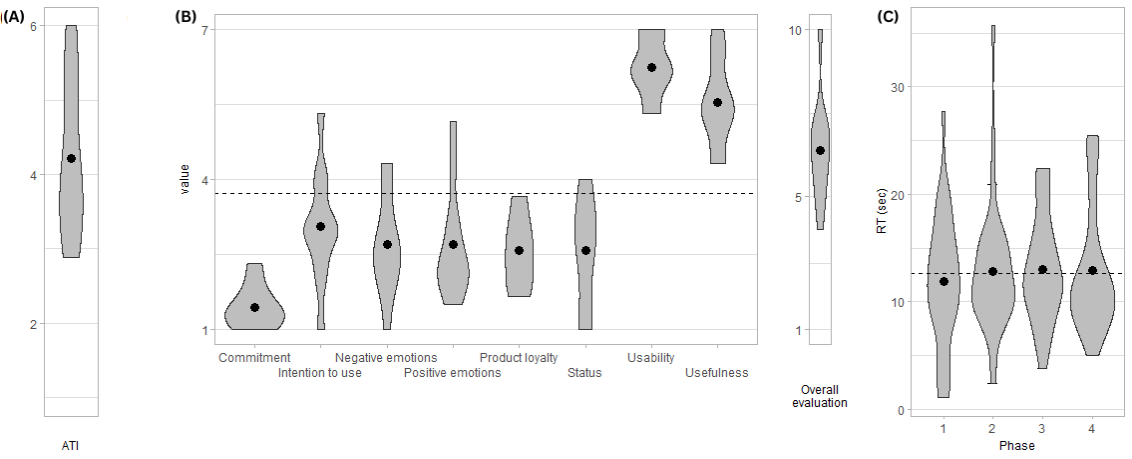
Violin plots of (A) Affinity for Technology Interaction (ATI; min=1, max=6), (B) score values across the constructs of modular evaluation of key Components of User Experience (meCUE; min=1, max=7, except for overall evaluation, which is min=1, max=10), and (C) reaction time across phases in study 0 (n=11). The dots represent the group mean; the dashed line represents the overall mean. RT: reaction time; sec: seconds.

Results are summarized in Figure 1.

#### Recruitment and Participants

We recruited 11 healthy individuals with a valid driver’s license via a web advertisement (ie, University of Zurich marketplace). One participant was excluded owing to simulator sickness. Thus, we included 10 participants (n=4, 40% female; n=6, 60% male) with an average age of 30.4 (SD 7.8; range 23-47) years and holding a license for 11 (SD 7.5; range 2-26) years, on average.

#### Technology Affinity Measure

Participants showed a mean ATI of 4.2 (SD 1; Cronbach α=.91), which is 70% of the maximal value.

#### Perception Measure

The meCUE (Cronbach α=.7) revealed a mean overall evaluation of 6.4 (SD 1.6), which is 64% of the maximal value. Moreover, the highest mean values were achieved for usability (mean 6.2, SD 0.6, 89%) and usefulness (mean 5.6, SD 0.9, 80%), whereas lower values were observed for commitment (mean 1.5, SD 0.4, 21%), positive emotions (mean 2.7, SD 1.1, 39%), negative emotions (mean 2.7, SD 1, 39%), intention to use (mean 3.1, SD 1.1, 44%), and product loyalty (mean 2.6, SD 0.7, 37%). A low value for negative emotions reflects a more positive evaluation.

To explain the perception results with the technology affinity measure, we correlated each meCUE construct with ATI. We observed a correlation between ATI and intention to use (*ρ*=0.70; *P*=.02). All the other correlations were not significant at the .05 level.

#### Compliance Measure

All the participants complied with the warning and stopped the car. Participants took 12.6 (SD 5.7) seconds on average.

#### Qualitative Feedback

Finally, some participants reported that the voice assistant spoke too fast to deliver information during a driving task without being distracting.

### Study 1: Assessment With Individuals With T1DM in Simulated Driving

**Figure 2:**
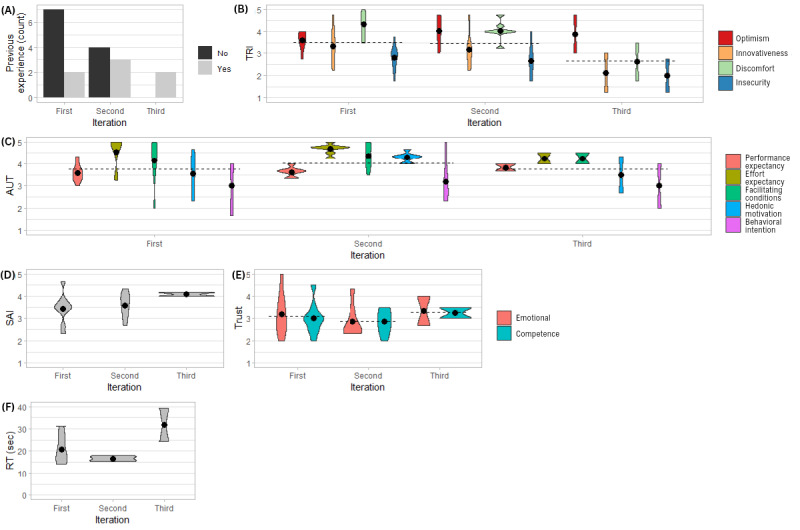
Violin plots of (A) count of previous experience, (B) score values across the constructs of Technology Readiness Index (TRI; min=1, max=5), (C) score values across the constructs of Acceptance and Use of Technology (AUT; min=1, max=5), (D) Session Alliance Inventory (SAI) scores (min=1, max=5), (E) Trust scores (min=1, max=5), and (F) reaction time across iterations in study 1 (n=18). The dots represent the group means; the dashed line represents the overall mean within an iteration. RT: reaction time; sec: seconds.

**Figure 3:**
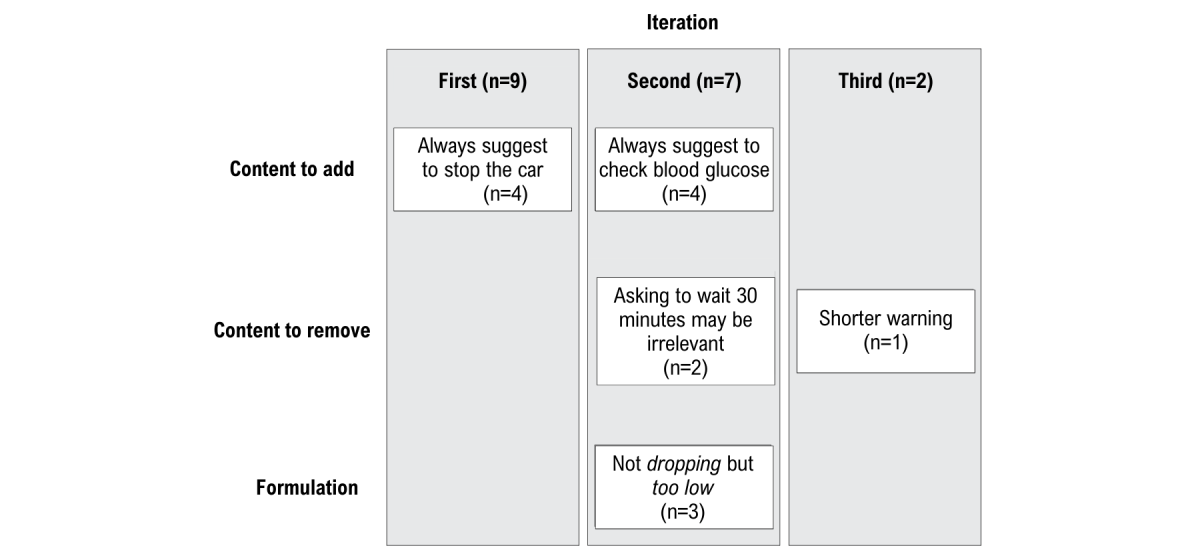
Thematic summary of participants' feedback in study 1 (n=18).

Results are summarized in Figures 2 and 3.

#### Recruitment and Participants

We recruited 20 patients with T1DM from the Department of Diabetes, Endocrinology, Nutritional Medicine, and Metabolism at the Bern University Hospital. Participants needed functional insulin treatment, good insulin self-management knowledge, a driver’s license, and active driving in the past 6 months. We excluded one participant owing to simulator sickness and one participant owing to technical errors in the warning delivery. This resulted in a total of 18 participants (n=6, 33% female and n=12, 67% male; mean age 31.4, SD 7, range 24-44 years; mean driver’s license age 13, SD 7.5, range 4.5-28.6 years). The first iteration had 9 participants, the second iteration had 7, and the third iteration had 2 participants. Although the last iteration’s sample size was small, it provided useful feedback to improve the warning for study 2.

#### Technology Affinity Measure

Seven participants had previous experience with an in-vehicle voice assistant (n=2, 28% in the first iteration; n=3, 43% in the second iteration, and n=2, 28% in the third iteration). TRI was 3.4 (SD 0.6, Cronbach α=.85), or 68% of the maximum. Specifically, TRI was 3.5 in the first and second iterations (SD 0.6 and 0.7, respectively) and 2.7 in the third iteration (SD 0.9).

#### Perception Measure

Perception averaged 3.9 out of 5 (SD 0.8, 78%) and remained stable across iterations. Average AUT (Cronbach α=.81) values were 3.8 (SD 1) in the first iteration, 4 (SD 0.7) in the second iteration, and 3.8 (SD 0.8) in the third iteration. Effort expectancy and facilitating conditions had the highest values across all iterations, whereas behavioral intention always had the lowest values.

SAI (Cronbach α=.79) averaged 3.7 out of 5 (SD 0.5, 74%) and increased slightly over the iterations, from 3.4 (SD 0.7) in the first iteration to 3.6 (SD 0.6) in the second iteration to 4.1 (SD 0.1) in the third iteration.

Trust (Cronbach α=.8) averaged 3.1 out of 5 (SD 0.7, 62%), was stable across constructs, and had the lowest values of the 3 perception measures. Trust averaged 3.1 (SD 0.8) in the first iteration, 2.9 (SD 0.6) in the second iteration, and 3.3 (SD 0.6) in the third iteration.

To explain the perception results with technology affinity, we tested the difference in perception (AUT, SAI, and Trust) between participants with and without previous experience with in-vehicle voice assistants. The means of all constructs, excluding facilitating conditions, were slightly higher for participants with previous experience. However, a 2-sided *t* test revealed no significant result (ie, *P*>.05).

We also correlated perception with TRI and observed a correlation between the optimism construct and performance expectancy (ρ=0.49; *P*=.04), behavioral intention (ρ=0.52; *P*=.03), and SAI (ρ=0.57; *P*=.01). All the other correlations were not significant (*P*>.05).

#### Compliance Measure

All the participants complied with the warning. In the first iteration, all participants answered *yes* or *no* to the receptivity check (“May I disturb you?”) and when asked if they had carbohydrates on hand. Five of the 9 participants answered yes to the latter question, although they did not. Two of those 9 participants stopped the car although they were not explicitly advised to do so. In the second iteration, all participants answered the prompts with yes and stopped the car as advised. One participant gave an affirmative *mhm* when asked, “May I disturb you?” during the hypoglycemic phase but were otherwise compliant. Because we used the Wizard-of-Oz method, the experimenter interpreted the affirmation. However, a current voice assistant might have interpreted it as an error. In the third iteration, both participants answered the prompts with yes and stopped the car. Across iterations, compliance took approximately 22 seconds. In particular, compliance took approximately 20 (mean 20.7, SD 6.2) seconds in the first iteration, approximately 17 (mean 16.7, SD 1.2) seconds in the second iteration, and approximately 31 (mean 31.7, SD 10.6) seconds in the third iteration.

#### Qualitative Feedback

Participants judged the voice warning as pleasant, simple, and as clear and efficient (n=15, n=11, and n=13, respectively). The topics for improvement are summarized in Figure 3. Note that these results are best understood when compared with Multimedia Appendix 1.

Given that Trust showed the lowest values in the first iteration, in comparison with the other perception measures, we decided to specifically ask participants, in our second and third iterations, what would help them trust the warning more. Of the 9 participants included in both the second and third iterations, 5 (55%) said they would just need to have a prolonged experience with the warning, whereas 3 (33%) said they would need to know what kind of data is used to infer that the driver is about to experience hypoglycemia. One participant did not know what would improve their trust.

### Study 2: Assessment With Individuals With T1DM in Real-World Driving Undergoing Hypoglycemia

**Figure 4:**
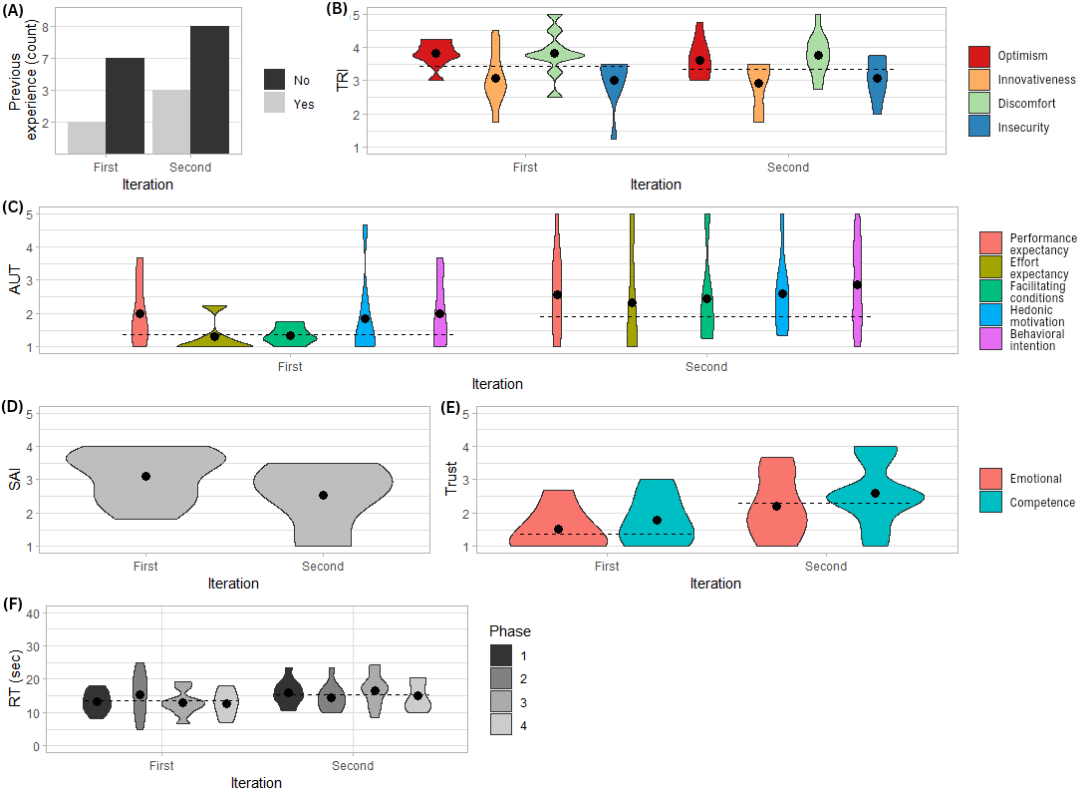
Violin plots of (A) count of previous experience, (B) score values across the constructs of Technology Readiness Index (TRI; min=1, max=5), (C) score values across the constructs of Acceptance and Use of Technology (AUT; min=1, max=5), (D) Session Alliance Inventory (SAI) scores (min=1, max=5), (E) Trust scores (min=1, max=5), and (F) reaction time across iterations in study 2 (n=20). The dots represent the group means; the dashed line represents the overall mean within an iteration. RT: reaction time; sec: seconds.

**Figure 5:**
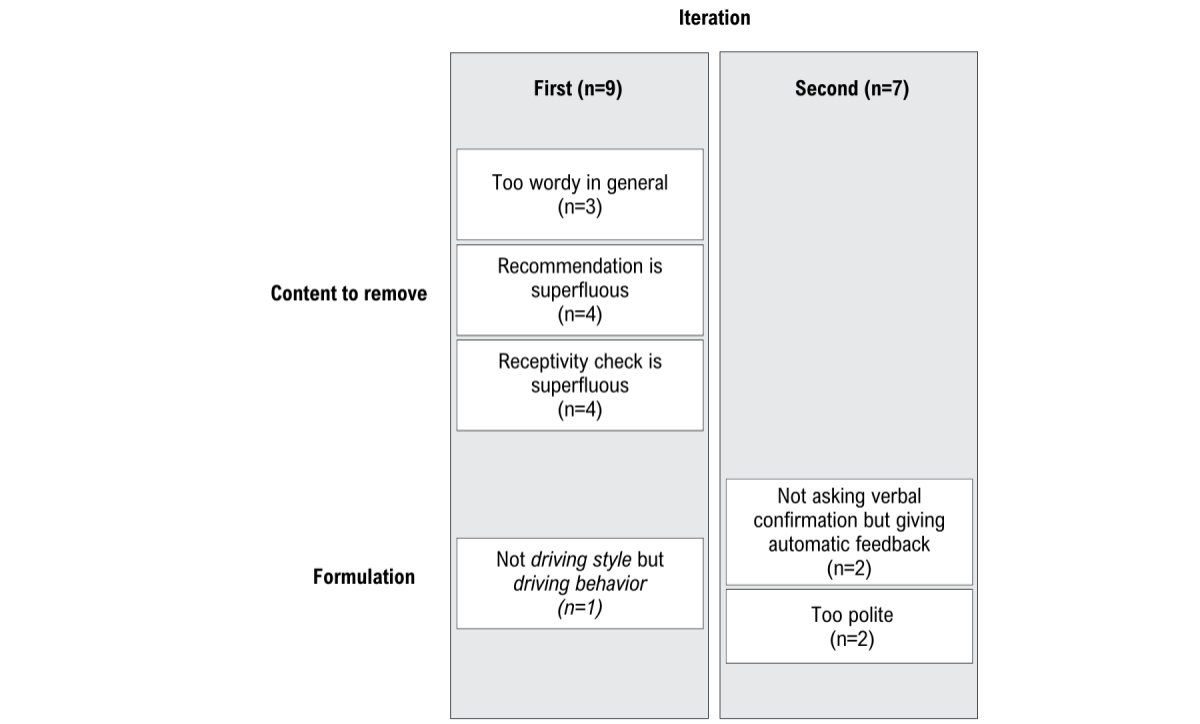
Thematic summary of participants' feedback in study 2 (n=20).

Results are summarized in Figures 4 and 5.

#### Recruitment and Participants

The recruitment procedure was the same as in study 2. We recruited 21 individuals, and 1 participant was excluded owing to data loss. Thus, we included 20 participants (n=3, 15% female and n=17, 85% male; mean age 40.9, SD 10.6, range 23-57 years; and holding a license on average since 23.7, SD 11.1, range 3.1-42.4 years). The first iteration included a sample of 9 participants and the second iteration included a sample of 11 participants.

#### Technology Affinity Measure

The pretest measurements revealed that 25% (5/20) of the participants had previous experience with an in-vehicle voice assistant (2 in the first iteration, and 3 in the second iteration), whereas TRI was on average 3.4 (SD 0.7; Cronbach α=.44), which is 68% of the maximal value. In particular, TRI was 3.4 in the first iteration (SD 0.8), and 3.3 in the second iteration (SD 0.7).

#### Perception Measure

The overall perception score was 1.7 out of 5 (SD 1.3, 34%). The results showed a slight increase in mean AUT (Cronbach α=.95) and Trust (Cronbach α=.85) values between the first and the second iteration, whereas SAI (Cronbach α=.80) showed a slight decrease. AUT also showed a considerable increase in SD. In particular, AUT values were on average 1.4 (SD 1) in the first iteration, and 1.9 (SD 1.6) in the second iteration; SAI was overall 2.8 out of 5 (SD 0.8, 56%). Values were on average 3.1 (SD 0.7) in the first iteration and 2.5 (SD 0.9) in the second iteration; Trust values were on average 1.4 (SD 0.8) in the first iteration, and 2.3 (SD 1.1) in the second iteration.

Similar to study 1, to explain the perception results with the technology affinity measure, we tested the difference in perception (ie, AUT, SAI, and Trust) among participants who had previous experience with in-vehicle voice assistants and those who did not. The means of all perception measures, excluding SAI, were consistently slightly higher in the second iteration. The means of all constructs, excluding SAI, were consistently slightly higher for participants who had previous experience with in-vehicle voice assistants. However, a 2-sided *t* test revealed no significant result (ie, *P*>.05). When correlating each perception measure with TRI, we observed a correlation between innovativeness and hedonic motivation (*ρ*=0.52; *P*=.02), a negative correlation between discomfort and behavioral intention (*ρ*=−0.46; *P*=.04), and a negative correlation between discomfort and competence trust (*ρ*=−0.45; *P*=.05). All the other correlations were not significant (*P*>.05).

#### Compliance Measure

All the participants complied with the warning. Two drives were excluded: one participant stopped once before the warning was delivered and data from one drive of one participant was lost. The results showed that the reaction time does not seem to vary across glycemic phases and, although minimal, there is a tendency for the reaction time to increase in the second iteration. Participants took 13.6 (SD 4.5) seconds in the first iteration and 15.5 (SD 4.1) seconds in the second iteration.

#### Preference for the Disclosure of the Triggering Cause

One participant was excluded because of data loss. The results showed that although 10 participants preferred when the warning was delivered with an explanation for the warning being triggered (in this case, driving behavior), 8 participants preferred it without the explanation. One participant stated that they would not use this in-vehicle voice warning either way.

#### Qualitative Feedback

In general, and similar to study 1, the participants found the communication style pleasant and efficient (n=4 and n=5, respectively). The topics for improvement are summarized in Figure 5. Note that these results are best understood when compared with Multimedia Appendix 1.

## Discussion

### Principal Findings

Most participants had not previously used an in-vehicle voice assistant, and technology affinity was similar across studies. In general, the voice warning elicited a positive perception, although the perception values were lower in the real-car study. In addition, participants complied with the warning in all studies, and reaction times were shorter in the real-car study than in the simulator study. Finally, the participants preferred the voice warning to be less verbose and prompt fewer interactions with the driver.

### Technology Affinity

Although we did not observe a significant effect on the perception of the warning, we suspect that the participants may have experienced a double novelty: using a voice assistant while driving and experiencing a warning from an in-vehicle voice assistant. Thus, future research should include a more balanced sample and compare the perception of a voice assistant–based warning with a standard warning (eg, an acoustic tone). Moreover, although we cannot directly compare ATI (used in study 0) with TRI (used in study 1 and study 2), we can observe that technology affinity was similar across studies. Although ATI showed a mean of 4.2 over 7 (60%), TRI showed a mean of 3.4 over 5 both in study 1 and study 2 (68%). The change in technology affinity measure was the result of an internal discussion between the coauthors, and we recommend the scientific community to use TRI in future research, as it is more widely used and focuses not only on the interaction but also on the general attitudes toward new technologies.

### Perception

We observed that AUT, SAI, and Trust values were higher in study 1 (simulated driving) than in study 2 (real-world driving). This evaluation might have been influenced by the driving setting. There can be 2 possible reasons. First, participants may have found the warning to be more distracting in the real car than in the simulator. However, research shows that drivers are more in control in real-world driving than in simulated driving [55]. Second, the technical difficulties in controlling the driver-assistant interaction owing to network slowdowns might have affected the user experience, and thus the perception measures. Future Wizard-of-Oz studies may account for this methodological weakness with a more accurate text-to-speech technology, avoiding remote control, and reducing interactions.

In addition, TRI seemed to have influenced behavioral intention (AUT) but did not consistently influence the other perception measures (ie, other constructs of AUT, SAI, and Trust). Thus, participants may have been excited about the potential of the voice warning, but they may not have been happy with the actual experience of using it.

### Compliance

The reaction times were short enough to ensure a timely reaction to the critical event. Blood glucose can change with a maximum rate of 0.22 mmol/L/min [56]. This means that someone driving with a normal glucose of 5.5 mmol/L might reach hypoglycemia (ie, 3.9 mmol/L) within a minimum of 7.5 minutes. Thus, although experiencing hypoglycemia while driving does not require an abrupt stop but rather a careful pullover maneuver and treating the condition, measuring reaction time provided an insight into the time required to take the first measure (ie, pullover). Interestingly, the reaction time was shorter in the real car (study 2) than in the simulator (study 0 and study 1). This difference may be attributed to the lack of traffic in study 2, which allowed the driver to pull over faster.

### Feedback

Although we aimed to keep the warning conversational, participants preferred a more direct notification of the problem without specific recommendations (eg, recommending waiting until the blood glucose is at its normal level) or polite formulations (eg, asking for permission to talk). To the best of the author’s knowledge, there was no in-vehicle voice warning at the time of the study, and we mostly relied on the guidelines of the Swiss Diabetes Association [10], while keeping the conversation as simple as possible. The participants’ feedback allowed us to improve the warning in this direction.

### Implications and Future Directions

#### Hypoglycemia Warnings

Reportedly, no research has been conducted for in-vehicle applications providing a hypoglycemia warning. However, smartphone apps for hypoglycemic events tracking have been investigated [57]. Although most of the research on glucose monitoring solutions conducted so far focused on diary apps rather than warning delivery, a pilot study on a smartphone-based hypoglycemia warning showed an improved hypoglycemia awareness and a reduction in daytime hypoglycemia [58] (other research is still in the phase of validation [59]). Future research should investigate such outcomes with an in-vehicle extension of this type of application.

#### In-Vehicle Warnings

Although there seems to be no related work testing the voice assistant of a private vehicle to deliver hypoglycemia warnings, there is a need for “driver-friendly” in-vehicle glucose monitoring solutions, expressed by the online community [25]. In particular, drivers with T1DM have contributed to the Nightscout Foundation [60], a nonprofit organization founded in 2014 and supporting open source technology for T1DM management, with the development of a data-sharing app, able to connect a car to a CGM, and display the glucose trends while driving on the dashboard of the private vehicle [25]. Moreover, there has been conceptual work manifesting the need for collaboration between automotive and medical industries to improve the safety of drivers with T1DM [24]. However, this work has not been followed by any implementation. Furthermore, no testing with the actual users has been conducted. Our work provides preliminary evidence, both in a simulated and a real-world environment.

Needless to say, recognizing hypoglycemia is only one part of glucose monitoring while driving; general imbalance of blood glucose (including hyperglycemia) can be problematic for the driver, if not dangerous [61]. Our work can be extended to hyperglycemia and, therefore, support further the safety of drivers with T1DM.

Finally, using the in-vehicle voice assistant to deliver a warning is compatible with current technology: not only are cars increasingly equipped with voice assistants [26,27] but also the automotive industry is aware of the relevance of using the upcoming “in-car proactivity*”* [62].

#### Warning Escalation

Our results showed 100% compliance in all 3 studies. This can only mean that the warning was clear enough for the participant to understand that it was time to pull over. That is, as all studies were run in a controlled setting, where an experimental team was present, and the participant knew they would be recommended to pull over eventually, we can safely assume that the experiments experienced a participant bias [63]. Thus, we cannot conclude that the warning was compelling enough to motivate the participants to comply (see the Limitations section). Nevertheless, the warning should be designed to allow for escalation, whereas in case the driver does not pull over in due time (eg, 2-3 min [56]) or explicitly rejects the warning, delayed reprompts with an increasingly severe tone would be delivered by the voice assistant (eg, “You are at risk of hypoglycemia. Please stop the car safely and check your blood sugar, then risk of hypoglycemia. Pull over now”).

#### Hypoglycemia Detection

Finally, in this paper, we focus on the interface between the hypoglycemia detection system and the driver, with the aim of visually distracting them as little as possible. Although the detection side is beyond the scope of this study, the designed warning is intended to be produced by a voice assistant built into the vehicle. Therefore, how a vehicle monitors blood sugar depends on the technology of the car. For instance, the aforementioned open source app displaying the glucose levels on the dashboard of a private vehicle [25] could be enhanced to connect with the in-vehicle voice assistant and use a voice warning instead of a visual one. Furthermore, research has been conducted on how to detect hypoglycemia from the car’s data [54] and from consumer-available wearable devices [64], with the argument that CGM devices can impose a social and financial burden on the individual.

### Limitations and Strengths

Despite our best efforts, this research has 3 main limitations.

First, the studies included a relatively small sample size. However, this study includes 3 feasibility studies (ie, a preliminary study with healthy individuals and 2 feasibility studies with individuals with T1DM), and the research presented in this paper is intended to be understood as an iterative development of a hypoglycemic warning. As such, this research aimed to pioneer the use of in-vehicle voice assistants for a driver health-related warning, rather than draw conclusions to be generalized to the population with T1DM. Thus, although we included a total sample size of 48 individuals, each feasibility study provides insight into the changes required by the users, and we provide the scientific community with an opening to the design of in-vehicle voice assistant–based health-related warning. Furthermore, previous studies on digital health systems used a similar sample size [65-67]. Thus, we believe that although the sample size does not allow drawing conclusions on the interaction of drivers with T1DM with in-vehicle hypoglycemia warnings, it still reports pioneer research.

Second, the studies were conducted over a short period. The participants had only a short-term experience with the warning. Perception and compliance may therefore be influenced by the novelty of such an experience, whereas perception may stabilize with repeated experience [68]. Future research should investigate the user experience of the warning in a longitudinal study. Third, these studies did not control for all potentially confounding variables related to real-world traffic and driver’s priorities. For instance, both simulator and real-car experiments involved disadvantages: while assessing the warning in a simulator allowed a controlled and safe experiment, such a setting remains artificial and lacks external validity. In contrast, while testing it in a real car increased the ecological validity of the human-machine interaction, it did not allow for as much traffic and speed variation as was possible in the simulator. Future research should investigate the effects of real-world traffic on the perception of the warning and compliance behavior. Moreover, receiving a warning in the presence of a team of experimenters may have influenced the participant’s verbal and behavioral responses; participants knew they would receive the warning sooner or later and had no reason not to follow it (eg, ignoring the warning because of being late for an appointment). In a real situation, drivers may not respond as expected or may even ignore the warning. Future research should test such a warning in a more ecological context, for instance, in a field study where the driver may not fall for a participant bias [69].

Finally, as we aimed to test a voice warning, our studies used a Wizard-of-Oz methodology to avoid problems related to natural language processing. Note that our studies were conducted in German-speaking Switzerland, where the German accents easily vary from region to region. As this aspect was beyond the scope of our research, we did not implement a working voice assistant or account for potential fallback intents triggered by the voice assistant’s failure to understand the user. Future research should push this research further and examine the potential danger of delayed treatment of hypoglycemia owing to the voice assistant’s natural language processing errors.

### Conclusions

Although hypoglycemia increases the risk of car mishaps [7,8], current solutions (eg, CGM and FGM) require visual human-machine interaction, which is inappropriate for an in-vehicle context. As voice assistants are increasingly present in private vehicles [26,27] and the European Commission fosters safety technologies inside the car [16], we propose to warn the driver of their critical health state through a voice assistant–based health warning. This paper reports on an iterative development and assessment of a hypoglycemia warning. In particular, we conducted in 3 studies: a preliminary study using a simulator with healthy participants, a test with individuals with T1DM in a simulator, and a test with individuals with T1DM in a real car. This gradual increase in authenticity in the experimental design allowed us to increase the ecological validity of our results while keeping experimental control. To the best of our knowledge, this is the first attempt of such a comprehensive feasibility assessment of an in-vehicle voice warning for hypoglycemia. Our results suggest that a voice warning can be useful, but that proactive behavior in voice assistants is still emerging and unfamiliar. We hope that these preliminary findings will foster future research to further develop in-vehicle hypoglycemia warnings.

## Appendix

Multimedia Appendix 1 Original (German) and translated version of the conversation flow of the hypoglycemia voice warning in study 1 and study 2.

Multimedia Appendix 2 Questions used in the semistructured interview about participants' experience with the warning conducted in study 1 and study 2.

Multimedia Appendix 3 Illustration of the procedure across studies.

## Abbreviations

ATI: Affinity for Technology Interaction
AUT: Acceptance and Use of Technology
CGM: continuous glucose monitoring
FGM: flash glucose monitoring
meCUE: modular evaluation of key Components of User Experience
RQ: research question
SAI: Session Alliance Inventory
T1DM: type 1 diabetes mellitus
TRI: Technology Readiness Index

## Data Availability

The data supporting the findings of this study are available from the last author, TK, upon reasonable request.
