# Supplementary material for: Effectiveness and User Perception of an In-Vehicle Voice Warning for Hypoglycemia: Development and Feasibility Trial
Source: JMIR Hum Factors. 2024 Jan 9;11:e42823. doi: 10.2196/42823 (PMC10813835; doi:10.2196/42823)
Supplement: Multimedia Appendix 1 [file humanfactors_v11i1e42823_app1.pdf]

Multimedia Appendix 1: Original (German) and translated version of the conversation flow of the hypoglycemia voice warning in Study 1 and Study 2

Bérubé et al. 2023

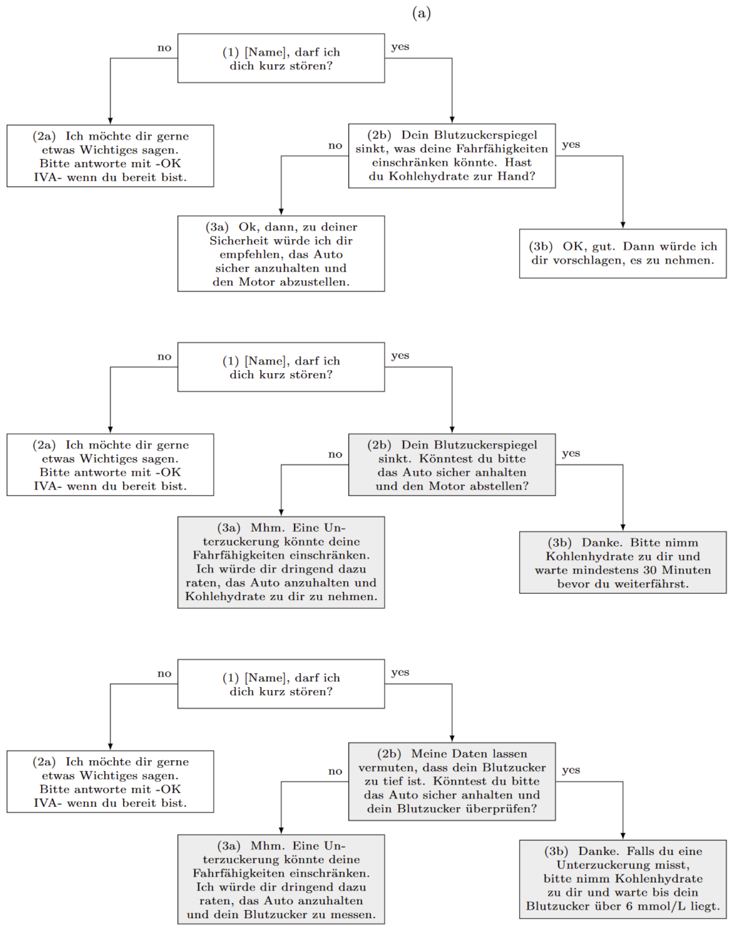

Supplementary fig. 1 Original conversation flow of the warning delivered in the first (a; n=9), second (b; n=7), and third (c; n=2) iteration of Study 1. Note: gray rectangles contain text that has been changed from the previous iteration.

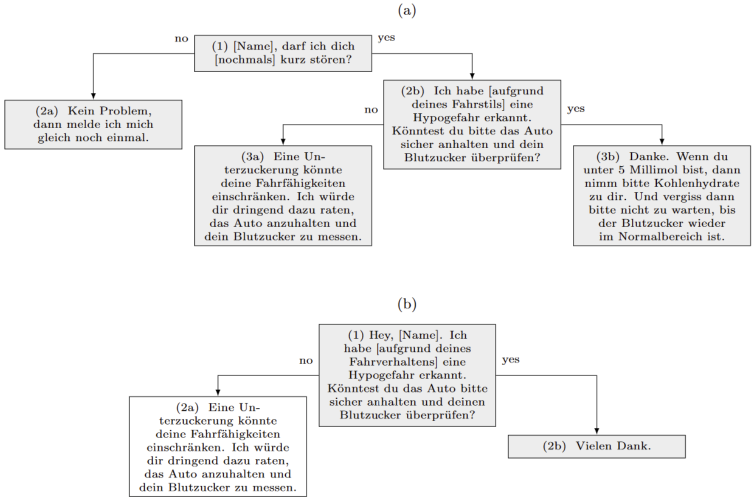

Supplementary fig. 2 Original conversation flow of the warning delivered in the first (a; n=9) and second (b; n=11) iteration of Study 2. Note: gray rectangles contain text that has been changed from the previous iteration.

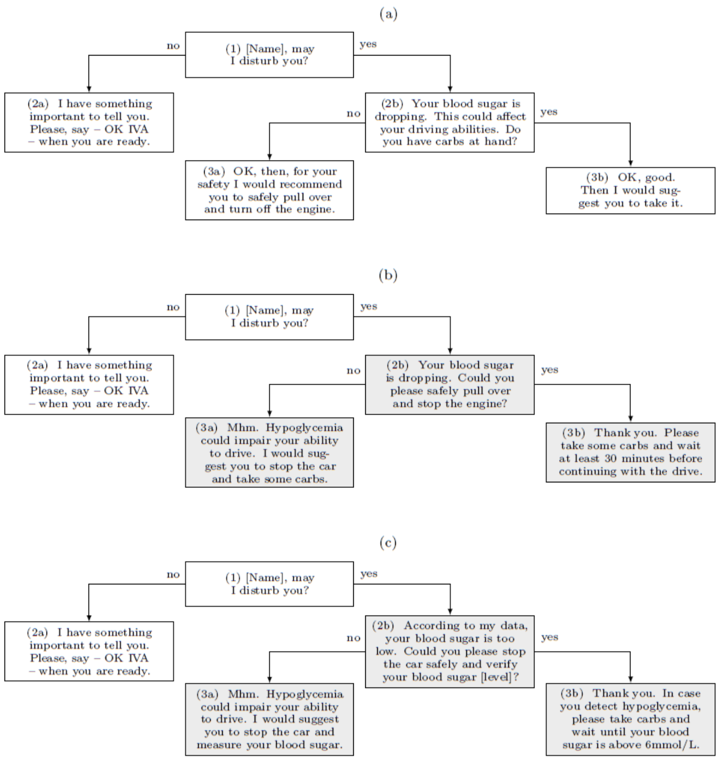

Supplementary fig. 3 Conversation flow of the warning delivered in the first (a; n=9), second (b; n=7), and third (c; n=2) iteration of Study 1. Note: gray rectangles contain text that has been changed from the previous iteration.

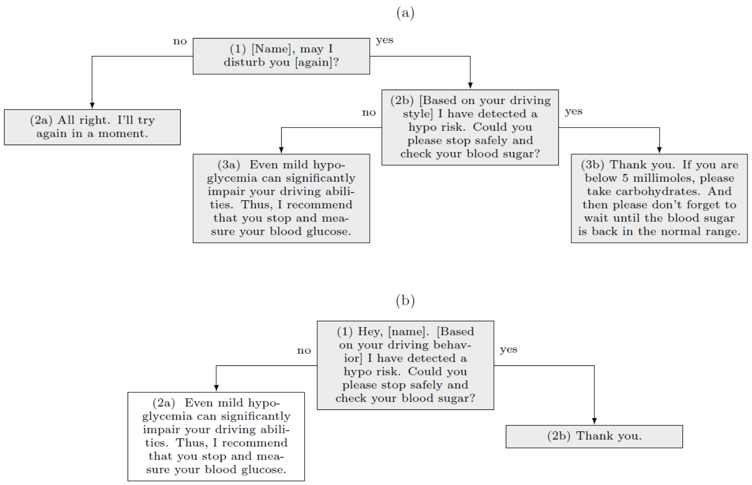

Supplementary fig. 4 Conversation flow of the warning delivered in the first (a; n=9) and second (b; n=11) iteration of Study 2. The text in square brackets was spoken in the variation with the explanation. Note: gray rectangles contain text that has been changed from the previous study or iteration.
