# Supplementary material for: Effectiveness and User Perception of an In-Vehicle Voice Warning for Hypoglycemia: Development and Feasibility Trial
Source: JMIR Hum Factors. 2024 Jan 9;11:e42823. doi: 10.2196/42823 (PMC10813835; doi:10.2196/42823)
Supplement: Multimedia Appendix 2 [file humanfactors_v11i1e42823_app2.pdf]

Appendix 2: Questions of the semi-structured interview about their experience with the warning conducted in Study 1 and Study 2.

Bérubé et al. 2023

Study 1

Original (German)	English translation
Wie haben Sie die Nachrichten vom Sprachassistent im Allgemein wahrgenommen? Was haben Sie darüber gedacht?	How did you perceive the notifications from the voice assistant in general? What did you think about it?
Was hat Ihnen gefallen?	What did you like?
Was hat Ihnen nicht gefallen?	What did you not like?
Was könnte an der Benachrichtigung geändert bzw. verbessert werden?	What could be changed or improved about the notification?
Was würde Ihnen helfen, (noch) mehr den Sprachassistent als Unterstützung für die Unterzuckerung beim Autofahren zu vertrauen?	What would help you trust (even) more the voice assistant as a support for hypoglycemia while driving?

Study 2

Original (German)	English translation
Wie würden Sie Ihre Gesamterfahrung mit dem Sprachassistent beschreiben?	How would you describe your overall experience with the voice assistant?
Was hat Ihnen bei der Verwendung den Sprachassistent am besten gefallen?	What did you like most about using the voice assistant?
Was hat Ihnen am wenigsten gefallen?	What did you like the least?
Was könnte noch verbessern werden?	What could still be improved?
Wie haben Sie die Benachrichtigung im Allgemein wahrgenommen?	How did you perceive the notification in general?
Was könnte [an der Benachrichtigung] geändert bzw. verbessert werden?	What could be changed or improved [about the notification]?
