# Supplementary material for: Effectiveness and User Perception of an In-Vehicle Voice Warning for Hypoglycemia: Development and Feasibility Trial
Source: JMIR Hum Factors. 2024 Jan 9;11:e42823. doi: 10.2196/42823 (PMC10813835; doi:10.2196/42823)
Supplement: Multimedia Appendix 3 [file humanfactors_v11i1e42823_app3.pdf]

Multimedia Appendix 3: Illustration of procedure across studies

Bérubé et al. 2023

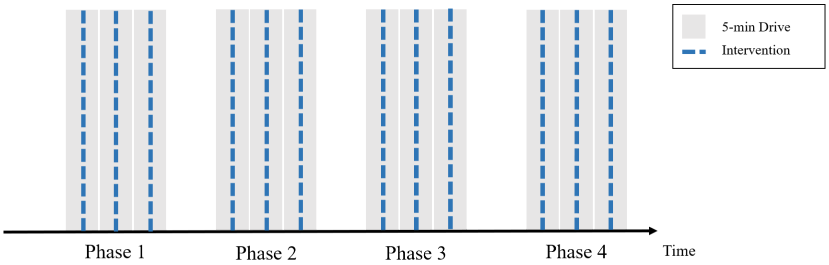

Supplementary fig. 1 Warning (t=100 sec or t=200 sec) in Study 0. This figure is shown to allow comparison across studies.

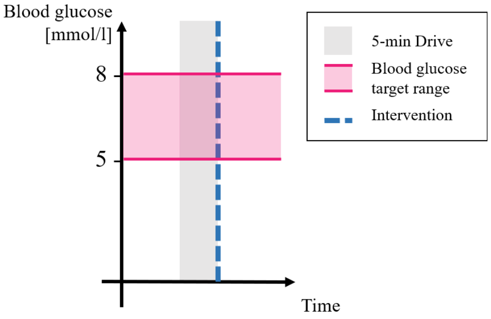

Supplementary fig. 3 Warning delivery procedure in Study 1. This figure is shown to allow comparison across studies.

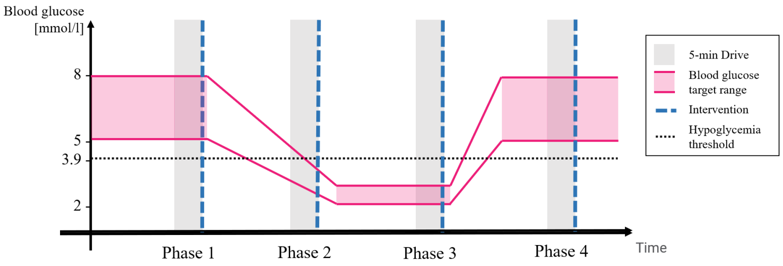

Supplementary fig. 3 Overview of the blood glucose manipulation and warning deliveries across the four driving phases in Study 2. This figure is shown to allow comparison across studies.
